# Essential Oils as Repellents against Arthropods

**DOI:** 10.1155/2018/6860271

**Published:** 2018-10-02

**Authors:** Mi Young Lee

**Affiliations:** Department of Medical Biotechnology, Soonchunhyang University, 22 Soonchunhyang–ro, Asan, Chungnam 31537, Republic of Korea

## Abstract

The development of effective and safe repellents against arthropods is very important, because there are no effective vaccines against arthropod-borne viruses (arboviruses) and parasites. Arboviruses and parasites are transmitted to humans from arthropods, and mosquitoes are the most common arthropods associated with dengue, malaria, and yellow fever. Enormous efforts have been made to develop effective repellents against arthropods, and thus far synthetic repellents have been widely used. However, the use of synthetic repellents has raised several concerns in terms of environmental and human health risks and safety. Thus, plant essential oils (EOs) have been widely used as an alternative to synthetic repellents. In this review, we briefly introduce and summarize recent studies that have investigated EOs as insect repellents. Current technology and research trends to develop effective and safe repellents from plant EOs are also described in this review.

## 1. Introduction 

Arthropod-borne viruses (arboviruses) are a public health concern worldwide owing to their association with several neglected tropical diseases [1, 2]. Thus, the discovery and development of effective and safe therapeutics against arboviruses remain a continuous goal. Arboviruses are transmitted to humans from arthropods and are categorized into several genera, including flaviviruses. Flaviviruses include yellow fever virus (YFV), West Nile virus (WNV), Japanese encephalitis virus (JEV), Zika virus (ZIKV), and dengue virus (DENV) [3].* Aedes aegypti* L. is the primary known vector of some viruses such as the dengue, yellow fever, chikungunya, and Zika viruses [4–6]. Other arthropods such as ticks, midges, flies, and fleas are also involved in the transmission of these viruses to humans [7, 8]. The most common mosquito-borne infectious disease in the United States today is caused by the West Nile virus (WNV), an arbovirus transmitted to humans by Culicine mosquitoes [9, 10]. There were 2,002 reported cases of WNV infections and 121 deaths across the US in 2017, according to the Center for Disease Control (CDC). However, there are no vaccines to prevent or medications to treat WNV. Dengue fever is by far the most rapidly expanding mosquito-borne viral disease, with an approximately 30 times increase worldwide in recent decades [11]. Approximately 40% of the population is at risk of infection, 50–528 million people are infected, and around 10,000–20,000 people die annually due to dengue [12, 13]. Ticks transmit Lyme disease, which is very common in the United States and Europe. Approximately 300,000 people per year in the United States and 65,000 people per year in Europe are affected by tick-associated Lyme disease according to the CDC [14, 15].

Unfortunately, however, there are no effective vaccines against these viruses and no specific drugs for inhibiting their propagation. As a consequence, controlling the spread of these neglected tropical diseases requires direct targeting of mosquitoes. Therefore, enormous efforts have been made to develop effective repellents and/or larvicides against arthropods. The synthetic repellent DEET (N,N-diethyl-3-methylbenzamide, formerly N,N-diethyl-m-toluamide) has been widely used. DEET is thought to work by blocking the insect's odorant receptors (olfactory receptor, ORx), which detect l-octen-3-ol, found in human breath and sweat, and not block the insect's ability to detect carbon dioxide [16]. However, the use of DEET has raised several concerns in terms of environmental and human health risks, especially in children [17]. Thus, plant essential oils (EOs) with good repellent properties and low toxicities to the environment and humans have been considered as an alternative to conventional synthetic insecticides [18, 19].

Plant EOs are volatile mixtures of organic compounds: in particular, mixtures of terpenoids and related aromatic compounds, which are secondary plant metabolites [20]. More than 3,000 EOs from various plants have been analyzed thus far, and approximately 10% of them are commercially available as potential repellents and insecticides [21, 22]. The chemical constituents of EOs are responsible for their antioxidative, antimicrobial, and pharmaceutical effects, as well as repellent and insecticidal effects [23–26].

This review focuses on recent studies that investigated EOs as insect repellents, exploring the relationships between the chemical ingredients and the repellent efficacy. Current technology and research trends to develop novel, effective, and safe insect repellents from plant EOs are also described. The articles discussed in this review were obtained via searching, until June 2018, major databases such as PubMed, Scopus, and Web of Science, with “essential oils,” “repellent/repellency,” and “arbovirus/arthropods” as keywords. A PubMed search for articles published from 1990 to June 2018, with terms related to repellent effect of EOs against arthropods, showed that there was a marked increase in the number of studies on finding natural alternatives to synthetic repellents, as evidenced by the drastic increase in the number of publications every 10 years (from 28 in 1990–1999, to 152 in 2000–2009, and to 412 in 2010–2018 September). The evidence obtained from these studies offers new perspectives to regulate hazardous arthropod vectors and control of the spread of severe neglected diseases among humans worldwide.

## 2. Essential Oils and Insect Repellents

A repellent is generally defined as a substance that discourages arthropods from landing or biting human skin [10]. The attractants for female mosquitoes include carbon dioxide and lactic acid present in sweat, and the resulting odor is recognized by chemoreceptors present in their antennae [27]. Insects detect specific scents via odorant receptors (ORx), which form complexes with coreceptors (Orco) that act as ion channels. When an odorant binds to an ORx, the Orco ion channel is opened, ultimately activating a sensory neuron that detects the odor [28]. Therefore, allosteric agonists and antagonists that target ORx and Orco could act as potential repellents by disrupting the odor-sensing activity in insects. Repellents, such as DEET, IR3535, and picaridin, have been proposed to act as olfactory agonists or antagonists, via modulating ORx activity, in the absence and presence of indole and octanol, which are specific to these ORx [29, 30]. There are various suggestions for the mode of action of insect repellency; however, the underlying mechanisms of insect repellency are not clear, and it is still a controversial topic [31]. Understanding the mode of action of insect repellents and how repellents modulate odor-sensing will allow us to design and develop better repellent formulations.

EOs are complex mixtures of volatile organic compounds from plants. The presence of monoterpenoids, sesquiterpenes, and alcohols has been proven to attribute to the repellent properties of EOs [32, 33]. In particular, citronellol, citronellal, *α*-pinene, and limonene are common constituents of many EOs exhibiting repellent effects [19, 34]. Recent evidence has shown that the odorant receptor neuron in a mosquito's antennal sensilla is activated by linalool, a naturally occurring terpene alcohol found in many flowers and spice plants, and by eucalyptol, a natural organic compound [28, 31]. The repellent screen platform based on odor-sensing might be new strategy for developing novel repellents or compounds with novel mode of action against arthropods.

## 3. Current Technologies for the Development of Effective and Safe Repellents Based on Essential Oils

### 3.1. Synergistic Interaction

Many methods have been described for the improvement of repellent efficacies of EOs. The most cited general method to increase the effectiveness of a repellent is to combine several EOs from different plants, leading to a synergistic effect [35–40]. The synergistic use of various components has been reported to provide a higher repellent activity than that obtained with single isolated components. A mixture of sesquiterpenes and monoterpenes present in different EOs was found to efficiently enhance the repellent effect, comparable to the effect of the sum of the individual components [37]. Mixtures of EOs derived from* L. cubeba*,* L. salicifolia,* and* M. leucadendron* led to a much stronger escape response by* Ae. aegypti* than each separate single EO [39]. EOs from* Cryptomeria japonica* were more effective against* Ae. aegypti* larvae than the combined major constituents including 16-kaurene and elemol. In addition, the minor compounds, including 3-carene, terpinolene, and *α*-terpinene, exhibited superior larvicidal activity against mosquito larvae [2, 40]. However, in some cases, the repellent effects of a mixture of synthetic pure compounds were not higher than those of a single compound. Moreover, synthetic blends formulated with major components of EOs had much lower repellent effects than their corresponding EOs [41, 42]. Interestingly, according to the reports on combined toxicity of Manuka, oregano, and clove bud EOs and their components against mosquito larvae, an antagonistic interaction between Manuka and clove bud EOs was reported, whereas a synergistic interaction between Manuka and oregano EOs occurred [21]. Carvacrol has been proposed to contribute to the synergistic interaction between Manuka and oregano EOs, whereas eugenol contributed to the antagonistic interaction between Manuka and clove bud EOs [21]. Taken together, these findings suggest that minor ingredients might play a major role in modulating repellent efficacy, suggesting the importance of compositional complexity in expressing repellency [43, 44].

### 3.2. Current and Emerging Technology

A variety of EOs have significant repellent effects; however, the effects tend to dissipate quickly due to their high volatility. EOs generally act in the vapor phase [45, 46], being active only for a short period of time. For example, citronella oil is highly volatile, and thus, insect repellents with citronella oil as the major component need to be reapplied every 20–60 minutes [47, 48]. The commercial citronella mosquito repellent contains up to 64% of the naturally occurring constituent p-menthane-3,8-diol (PMD), which is primarily responsible for the efficacy and protection against insects and biting arthropods [10, 31]. The drawback of the short protection time could be improved via formulation technology development, by retaining the active components on the skin for longer periods. Cream-based formulations and polymer mixture-based formulations led to an increase in the repellent effect. A topical formulation of lemon grass oil prepared in petroleum jelly base resulted in long-lasting protection, without side effects [49].

Moreover, microencapsulation resulted in an increase in repellency duration [50, 51], via controlled release of the EOs. Microencapsulation of* Z. limonella* oil in glutaraldehyde crosslinked gelatin enhanced the repellent effect against mosquitos [52]. Microcapsules containing thyme oil prepared by using a melamine–formaldehyde prepolymer showed sustained release properties and long‐lasting repellency of the encapsulated EO [53].

Interestingly, an increase in repellent efficiency was also reported when fixative agents including vanillin, liquid paraffin, and salicyluric acid were used. The most widely used fixative agent is vanillin [54], and the duration of repellency against arthropods including* Ae. aegypti* by EOs was notably enhanced when vanillin was mixed with the EOs. Upon adding vanillin, the protection time of* Zanthoxylum piperitum* oil and Citronella oil was extended to 2.5 h and 4.8 h, respectively [53]. In addition, microencapsulation or nanoemulsification combined with vanillin treatment enhanced the effects and protection times of repellents. The success of geranium EOs against* Culex pipiens *can be attributed to the microemulsion formulations prepared based on nonionic surfactants such as Tween 80 [55–58].

Currently, nanotechnology is extensively used to prepare repellents with EOs for better efficacy [59]. Nanoparticle fabrication by using plant components as reducing and stabilizing agents has several advantages compared with conventional methods. Nanoparticles can penetrate through the exoskeleton and interact with functional biomolecules, resulting in the disturbance in membrane permeability and proton motive force [60, 61]. The size, shape, and efficacy of nanoparticles against arthropods vary depending on the plant sources used as reducing and stabilizing agents. For example, silver nanoparticles containing neem were mostly spherical [62, 63], whereas silver nanoparticles fabricated using leaves from* Carissa spinarum *were cubical [64–66]. In addition, the use of nanotechnology for EO delivery could reduce the cost, steps for development process, and risks associated with pressure, temperature, and energy [67].

Recently, an EO-based polymeric patch, embedded into ethylcellulose and polyvinylpyrrolidone (PVPK-30) polymers, was developed, and the physicochemical properties and efficiency of oil release against* A. albopictus* were assessed [56]. Notably, the EO repellent patch formulation was found to be safe in animal models in terms of respiratory, hematological, and biochemical parameters. Thus, the matrix-type patch formulation of the repellents seems to be an effective approach to deliver repellents into the surrounding environment. In addition, the mosquito repellent activity of cotton, functionalized with inclusion complexes of *β*-cyclodextrin citrate and EOs, was also measured against* Anopheles stephensi*. Peppermint and lavender were effective as potential repellents in cotton with inclusion complexes of *β*-cyclodextrin [68]. Currently, eco-friendly and cost-effective EOs with antiviral capacity against arboviruses such as Ross River virus (RRV) are being studied [69]. Three EOs from* Cymbopogon citratus *(CC),* Pelargonium graveolens *(PG), and* Vetiveria zizanioides *(VZ) with repellent activities were evaluated for antiviral effects, in terms of prevention of viral entry, using a wild-type RRV-T48 strain, and viral replication, using a recombinant RRV expressing* Ren *luciferase.

Codelivery of piperonyl butoxide (PBO) and 35 EOs along with permethrin, a common synthetic pyrethroid, was reported to determine whether the EOs could increase the effect of permethrin against* Ae. aegypti *and* Anopheles gambiae *[70]. The results suggested that the EOs elevated the efficacy of permethrin than that of PBO and thus can be used as a natural alternative to classic chemical synergists such as PBO [70]. Moreover, diverse EOs and EO terpenoids have been shown to provide synergistic effect with permethrin against arthropods [71–73], although the underlying mechanism is not clear. Further investigations are required to clarify the role of individual terpenoids and their capability to upregulate permethrin efficacy.

In addition, quantitative structure-activity relationships (QSARs) could be applied for novel repellent development. QSAR is based on the differences in the physicochemical properties, including lipophilicity, shape index, and electrostatic nature, of terpenoids, which serve as a backbone for the synthesis of novel repellents. The QSAR approach might contribute to a better understanding of structural properties of terpenoids suitable for repellency, as well as predicting the repellent efficacy of other terpenoids [74, 75]. Recently, the larvicidal activity of fifty constituents of various EOs was evaluated against* Culex quinquefasciatus *Say using QSAR models in order to identify molecular and structural properties for larvicidal activity. In this study, molecular docking on the SCP-2 protein by *α*-humulene and *β*-caryophyllene was proposed to clarify the mechanism of action. This can be useful for designing and synthesizing new larvicidal agents [76]. Currently, a new technology has been applied to measure the toxicity of plant phytochemicals, using random amplified polymorphic DNA-polymerase chain reaction (RAPD-PCR). This technique is used for large-scale analysis of multiple samples for measuring the effects of genotoxic chemicals. Thus, RAPD-PCR technique can be used to evaluate the toxicity of repellents in biological systems by studying genomic DNA damage and mutation. To evaluate the toxic effects, as well as genotoxic hazards, of three plant oils and a recommended insecticide (Chlorpyrifos) against* R. dominica *adults, RAPD-PCR has been previously used [77].

Recent rapid progress in biotechnology has led to a massive increase in the use of multiomics tools including genomics, proteomics, and metabolomics data. However, these tools have not been utilized to find novel and more effective insect repellents. In fact, as the number of disease-causing viruses is increasing, it is necessary to use these data to a maximum extent to develop highly effective and useful repellents [32]. EOs with diverse constituent chemicals show severe complexities, and thus new technological approaches are required to understand the complex and diverse properties of EOs to treat neglected diseases [33, 34].

Interestingly, gene expression studies associated with molecular pathway analysis and transcriptome analysis using RNA-seq have been conducted to evaluate the effects of a large panel of EOs on the transcriptional landscape of human cells [78, 79]. In addition, emerging application of microarray-based gene expression profiling and pathway-based testing of chemicals [60, 80–82] could be utilized for toxicity pathway-based tests of new types of EOs against insect vectors. Ingenuity Pathway Analysis was used to analyze the effects and the potential toxicities of rosemary oil and orange oil [83], which can be applied to develop insect repellents. The signaling pathway-based evaluation and the assessment of potential risks of the phytochemicals, based on the representative chemical components associated with beneficial effects, were achieved by using DNA microarray assay [84]. The pathway-based evaluation of the beneficial effects and potential risks by means of multiomics technology seems to be promising for finding novel EO-based repellents. The emerging application of novel technology has entered a new era associated with recent innovations in insect repellent development.

## 4. Toxicity and Safety of EOs as Repellents against Arthropods

In general, plant EOs are recognized as safe; however, some of them also cause side effects such as skin irritation, mainly due to the constituents present in the plant EOs, limiting their extensive usage [85, 86]. Natural products are not always safer than synthetic ones, and some might cause adverse reactions. Moreover, sometimes, the available toxicity and safety information is limited and contradictory [31, 87]. The repellent effects of EOs from* Rosmarinus officinalis*,* Dacrydium franklinii,* and* Melaleuca bracteata* were found to be promising, but were unsuitable for human use because they caused skin irritation, contact dermatitis, and asthma [18, 88–90]. At present, synthetic repellents are more widely used than EOs, although this raises several concerns related to environmental and human health.

In the United States, the FDA tests and approves topical insect repellants such as DEET for use [10]. Further, citronella, lemon, and eucalyptus oils are common insect repellents and are registered by the EPA and have been approved for topical use in humans. PMD (p-menthane-3,8-diol), proven safe to human health, is the only plant-based repellent advocated by the CDC for public use. 2-Undecanone, a natural compound from the wild tomato plant* Lycopersicon hirsutum*, is a biopesticide product with lesser toxicity than conventional pesticides. BioUD formula is the only EPA-registered insect repellent containing 2-undecanone [91]. Natural EOs such as lemongrass, citronella, cedar, peppermint, lavender, and geranium oils are exempted from the Environmental Protection Agency registration. The duration of effectiveness of these oils is estimated to be between 30 minutes and 2 hours. An overview of comprehensive properties of commercially available repellents is described in Table 1. The general properties, toxicity, and safety of major conventional repellent EOs are described below.

*Citronella*. Citronella (3,7-dimethyloct-6-en-1-al), from* Cymbopogon citratus*, or lemon grass oil, has been used as a topical insect repellent in children and other sensitive populations under the US EPA (Environmental Protection Agency) guidance with appropriate precautionary labeling [96, 97]. Moreover, the US FDA (Food & Drug Administration) considers citronella oil as GRAS. Citronella can deter mosquito biting for 2 hours, showing lesser effectiveness than DEET in terms of duration of protection against mosquito bite. However, recently, citronella is not being used as an insect repellent in Canada and Europe. The Canadian regulatory concerns with citronella as an insect repellent are due to a lack of safety data and the presence of methyl eugenol [98]. Methyl eugenol is categorized as a group 2B substance, possibly carcinogenic to humans, by the IARC (2013), as there is sufficient evidence in experimental animals indicating carcinogenicity, although no data are available for humans. In addition, methyl eugenol is “reasonably anticipated to be a human carcinogen” by the US National Toxicology Program (NTP), due to its carcinogenicity in other rodents, but not in rats. In addition, citronella oil is regarded as a category 3 substance, with some safety concerns regarding the presence of methyl eugenol, and is not sold as an insect repellent within the EU since 2006 [98].

*Clove Oil*. Clove oil, from* Syzygium aromaticum*,* Eugenia caryophyllata, *or* Eugenia aromaticum,* has been widely used in food, cosmetics, and medicines, as well as in insect repellents. However, some reports have raised concerns that methyl eugenol, one of the trace components of clove oil, could be a carcinogen. The major components of clove oil are eugenol, eugenol acetate, and caryophyllene. Clove oil was approved by the FDA under its Code of Federal Regulations (CFR) 21 as generally recognized as safe (GRAS) and can be added directly to human food (21 CFR 184.1257) in both natural and synthetic forms. In addition, clove oil was approved for use in dentistry as an analgesic and in dental cements, as a fragrance in personal care and aromatherapy products, and in transdermal drug delivery systems by the FDA. The US EPA also categorized clove oil under the Section 25(b) list of Minimum Risk Pesticides, being exempt from most pesticide registration requirements, including extensive toxicity testing. However, the US National Library of Medicine reported some side effects of clove oil, including skin irritation, headache, and increased bleeding due to decreased blood clotting. In conclusion, eugenol or clove oil use may be safe, and its carcinogenic potential or other adverse effects need to be established scientifically.

*PMD*. PMD (p-menthane-3,8-diol) is a major repellent ingredient extracted from the leaves of lemon eucalyptus,* Eucalyptus citriodora,* or* Corymbia citriodora*. It can be chemically synthesized for use in commercial repellents. Citronellol, limonene, and linalool were found in the extracts from eucalyptus, together with PMD. PMD is a highly effective and long-acting mosquito repellent, similar to DEET. Moreover, PMD exhibited better protection against ticks than DEET, by inhibiting attachment and blood-feeding of tick vectors associated with Lyme disease and Rocky Mountain spotted fever [10].

EPA has registered the oil of lemon eucalyptus (OLE) or PMD as a biopesticide repellent, under repellents derived from natural materials in 2000. However, “pure” oil of lemon eucalyptus, EO not formulated as a repellent, is not recommended as an insect repellent by the EPA, as there are no studies on its safety and efficacy. PMD is categorized as an eye irritant, but not classified as a skin sensitizer. PMD is classified under Toxicity Category I as a technical product and under Toxicity Category II as an end-use product. Products containing PMD must carry a “warning” label, because it may cause eye irritation. Acute toxicity studies on PMD have shown low toxicity, but there are little epidemiologic data on the effects of PMD. PMD, as an active ingredient, is classified as GRAS by the EPA, is used to flavor foods and medicines, and is a component of many consumer products. The FDA has recommended that PMD not be used in children under 3 years of age. The Center for Disease Control and Prevention recommends the use of PMD products as they prevent mosquito bites.

*Permethrin*. Permethrin is one of the most common synthetic Type I pyrethroidal neurotoxic insecticides, obtained from the dried flowers of* Chrysanthemum cinerariifolium*. Its main mechanism of action is axonal sodium channel depolarization causing repetitive nerve impulses as a neurotoxin [99]. High-dose pyrethroids can affect gamma-aminobutyric acid (GABA)-gated chloride channels, resulting in seizures with severe type II poisoning. Permethrin showed increased acute toxicity against cold-blooded organisms including insects compared to that against warm-blooded organisms including mammals. It showed minimal topical absorption with an approximate half-life of 70 days. Permethrin is recommended to be used as an insect repellent on clothing only and direct skin contact should be avoided. Permethrin-treated clothes were effective against insects for 2 weeks to 6 months (5–20 detergent washings). It can also be applied to bed nets and beddings with high-level protection against mosquitoes, ticks, and other insects [10]. Human exposure to large doses of permethrin may cause genotoxicity and immunotoxicity in humans and farm animals. However, it is classified by the EPA as a likely human carcinogen, based on a mouse reproduction test.

## 5. Conclusions and Future Perspectives

The wide use of synthetic repellents against arthropods has raised some issues on safety and health risks to humans and the environment. Thus, EOs from plants could be considered as alternatives to synthetic repellents. At present, the development of natural product-based repellents with more effective and long-lasting protection is required. The recent rapid progress in the field of biotechnology might ensure the development of natural repellents with improved repellency, long-lasting protection, and enhanced safety. In particular, current formulation technology and nanotechnology might accelerate the emergence of novel and effective EOs with long-lasting repellency effects. Moreover, toxicological studies associated with multiomics can also be applied to find novel repellents, monitor target exposure to repellents, track cellular responses to different doses, assess mechanisms of action, and predict health risks associated with existing and newly developed EOs.

## Figures and Tables

**Table 1:** An overview of commercially available repellents of synthetic and natural origins.

Repellent	Active compound	Repellency	Safety/Toxicity	Origin
DEET	*N*,*N*-diethyl-3-methyl-benzamide. (*N*, *N*-diethyl-m-toluamide) 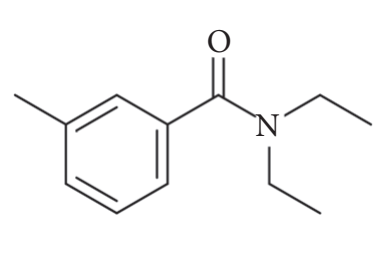	23.8% (6.65%) DEET provides about 5 (2) hours of protection against mosquitoes [92]	Potential neuro toxicity if applied under sunscreen. EPA toxicity category III (slightly toxic)	

Picaridin	(2-(2-hydroxyethyl)-1- piperidine-carboxylic acid 1-methylpropyl-ester) 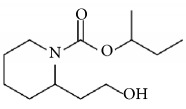	Picaridin 20% works as well as DEET 20% [93]	Possible skin irritation. EPA toxicity category III (slightly toxic)	

Permethrin	(3-phenoxybenzyl (1RS)- cis, trans-3-(2, 2- dichlorovinyl)-2, 2- dimethyl-cyclo-propanecarboxylate) 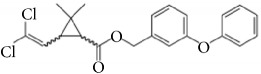	Permethrin medication is applied to skin as a cream or lotion. Permethrin repellents should be used on clothes.	Not useful on skin. Possible skin irritation. (EPA, likely human carcinogen)	*Chrysanthemum* spp.

PMD (Oil of lemon eucalyptus)	(p-menthane-3, 8-diol) 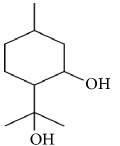	PMD is as effective as DEET when used in like quantities. PMD provides about 2 hours of protection against mosquito bites [94]	Potential skin irritation in atopic individuals. EPA toxicity category I(highly toxic)	Lemon eucalyptus

Citronella	(3, 7-dimethyloct-6-en-1-al) 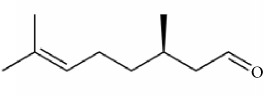	The complete protection time (CPT) of DEET (360 min) was much longer than the CPTs of citronella (10.5 min) from mosquitoes [95]	Potential eye and skin irritation and allergies for Ceylon type EPA toxicity category IV (practically non-toxic)	*Cymbopogon* spp.
